# Application of Tannic Acid and Fe^3+^ Crosslinking-Enhanced Pectin Films for Passion Fruit Preservation

**DOI:** 10.3390/foods12183336

**Published:** 2023-09-06

**Authors:** Jun Yang, Wenjin Cai, Mohammad Rizwan Khan, Naushad Ahmad, Zhengke Zhang, Lanhuan Meng, Wanli Zhang

**Affiliations:** 1 School of Food Science and Engineering, Hainan University, Haikou 570228, China; 2 Department of Chemistry, College of Science, King Saud University, Riyadh 11451, Saudi Arabia

**Keywords:** crosslinking, film properties, pectin films, preservation applications

## Abstract

In this work, the role of tannic acid (TA) and Fe^3+^ in crosslinking pectin (PE) to enhance its physicochemical properties was investigated. Specifically, PE/TA/Fe^3+^ composite films were prepared using the solution casting method, and the UV-blocking properties, transparency, water content, physico-mechanical properties, antioxidant properties and degradability of the PE composite films were investigated. The microstructure of the PE composite films and the interactions between the contained components were analyzed using FTIR, X_crystal diffraction and SEM scanning electron microscopy. The results showed that the addition of TA and Fe^3+^ can significantly improve the UV barrier properties and antioxidant properties of PE films. Meanwhile, Fe^3+^ could form a metal phenol network with TA and crosslink with the PE film, which makes the structure of the PE film denser and thus significantly reduces the water vapor permeability of the PE film. In addition, this work also indicated that the PE composite coatings have a favorable preservation effect on passion fruit, which leads to the lowest weight loss rate and wrinkle index of the passion fruit within 7 days of storage and shows good appearance quality and commercial value. This work indicates that the addition of tannic acid and Fe^3+^ significantly improved the mechanical and barrier properties of pectin films, and the composite pectin coating extended the shelf life of passion fruit.

## 1. Introduction

With the rapid development of the social economy, people’s living standards have gradually improved. Consequently, more and more individuals are becoming concerned about environmental protection, food safety and packaging. Traditionally, petroleum-based plastic products have been commonly used as food packaging materials. However, these plastic products suffer from the drawback of being non-biodegradable. The global production of plastic resin continues to increase annually, while the recycling rate remains below 5%, leading to a significant rise in global plastic waste, imposing a serious burden on the environment [1]. Furthermore, microplastic particles present in petroleum-based plastic products can easily migrate into food items, posing a severe threat to consumer health. As a result, the development of green, natural and biodegradable food packaging films is of utmost importance [2]. Presently, various natural polymers, such as polysaccharides, proteins and lipids, are being utilized to produce biodegradable food packaging films due to their favorable film-forming properties [3,4,5].

Pectin (PE) is a group of D-galacturonic acids found in the epidermis of plants and is the main component of plant cell walls. According to previous studies, it can be divided into high-methoxyl PE with a carboxyl esterification rate of more than 50% and low-methoxyl PE with a carboxyl esterification rate of less than 50% [6]. In plant-derived foods, PE is often discarded as food by-products in large quantities, resulting in resource waste and environmental burden. The excellent film-forming properties of PE have been highly regarded by scientists in the preparation of edible coating films and food packaging films [7]. However, the strong hydrophilicity of pure PE films results in poor water resistance, and their mechanical properties are not sufficient for practical food packaging applications [8]. To address the above limitations, many studies have utilized crosslinking techniques to improve the physicochemical properties of pure PE films. A previous study from our group has shown that PE can chelate cations such as calcium and iron ions to produce a network structure with enhanced water resistance, and the PE and iron ion crosslinked films showed enhanced UV barrier properties, water vapor barrier properties and tensile strength [9].

Tannic acid (TA) is a natural phenolic acid widely present in plants, and its molecular structure consists of gallic acid and glucose linked by a fatty ester bond [10]. It is abundant in nature and offers the advantages of low cost and favorable biocompatibility. TA appears as a brownish-yellow solid powder at room temperature and contains a polyphenolic structure that crosslinks with polymers, imparting excellent antimicrobial and antioxidant effects. In particular, TA can bind metal ions via o-dihydroxyphenyl groups, thereby preventing bacterial uptake of metal ions and enhancing their antimicrobial activity [11]. Furthermore, TA exhibits a strong crosslinking effect, and when crosslinked with polymers, it significantly enhances their mechanical properties. For example, the incorporation of TA into casein films increased the tensile strength of the films by about 55.17%. This enhancement was attributed to the crosslinking effect of TA, which improved the network structure of the casein films, thereby increasing their mechanical strength [12]. Moreover, TA can form a metal–phenol supramolecular network structure with Fe ions, which helps improve the inhomogeneous gelation structure that can result from direct crosslinking between TA and the polymer [13]. It has been reported that the addition of Fe^3+^ and TA to chitosan films can increase the mechanical properties of chitosan films by about 3 times and reduce the water vapor transmission rate by about 50.38% [14]. Therefore, the use of the multi-crosslinking system formed by TA, Fe^3+^ and PE may be an effective strategy to improve the properties of PE films.

Passion fruit is a fruit with a unique aroma, rich in amino acids and nutrients needed for the human body, which can play a role in healthcare and tranquility and is favored by consumers. However, passion fruit is a typical respiratory leap fruit, which is susceptible to microbial and environmental influences after harvesting, and loses its commercial value due to water loss and crumpling within a short period of time. In recent years, many studies have been conducted to develop different preservation techniques to preserve passion fruit, such as chemical fungicides, low-temperature storage and functional coatings. A recent study developed a chitosan-based coating for the preservation of passion fruit and demonstrated that tannic acid incorporated into the chitosan coating significantly retarded the senescence and inhibited the respiratory metabolism of passion fruit, thereby extending the shelf life of passion fruit [15].

To the best of our knowledge, there are no relevant reports on the double crosslinking between tannins, Fe^3+^ and PE to enhance the mechanical and barrier properties of PE films. Therefore, this study focuses on the development of PE biocomposite membranes using TA and Fe^3+^ and explores the physical and barrier properties of PE composite membranes through microstructural analysis (SEM), FTIR analysis and X-ray diffraction (XRD) analysis. Furthermore, the effect of the PE composite film on the freshness of passion fruit was investigated, and the appearance, weight loss rate and wrinkle index of coated passion fruit were determined.

## 2. Materials and Methods

### 2.1. Materials

Pectin (PE) was obtained from Zhengzhou Feiman Biotechnology Co., Ltd. (Zhengzhou, China). Tannic acid (TA) and glycerol were obtained from Fruit & Vegetable Team Lab. Passion fruits were purchased from Baoting Hesheng Agricultural Development Co., Ltd. (Hainan, China), and passion fruits of the right size, free of mechanical damage and disease, were selected and picked from the orchard, transported to the laboratory of Hainan University within 24 h, and then cleaned and dried to be ready for the use.

### 2.2. Preparation of PE/TA/Fe^3+^ Composite Film

PE composite films were prepared using a solution casting method following the procedure described by Zhang et al. [14]. Firstly, 3 g of PE was weighed in a 250 mL beaker, and then 100 mL of distilled water at a temperature of 70 °C was added to dissolve the PE. The mixture was stirred for 2 h until a 3% PE film solution was obtained. Next, 0.9 g of TA was weighed and dissolved in 30 mL of distilled water to create a 30% TA solution (based on PE mass, *w*/*w*). Similarly, 0.003 g of ferric chloride was weighed and dissolved in 20 mL of distilled water to create a 0.1% solution of Fe^3+^ (based on PE mass, *w*/*w*). The TA solution and Fe^3+^ solution were slowly added to the PE solution with stirring, using a homogenizer, to form a 2% PE composite film. Additionally, 30% glycerol (based on PE mass, *w*/*w*) was added as a plasticizer. The resulting PE/TA/Fe^3+^ (pectin/tannic acid/Fe^3+^) composite film solution (20 mL) was poured into polystyrene plastic Petri dishes with a diameter of 90 mm. The dishes were then dried in a blast oven at 60–70 °C for 6 h. Similarly, pure PE films and PE/TA (pectin/tannic acid) films were prepared at a concentration of 2%. The specific programs are shown below.


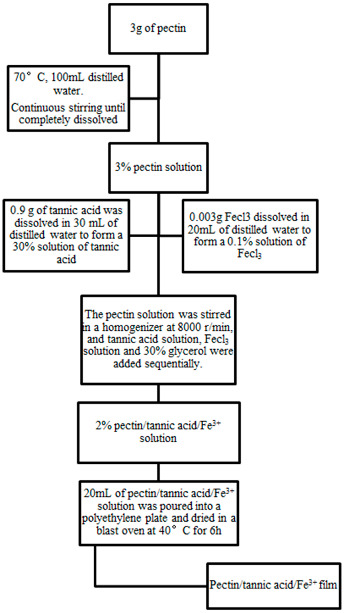


### 2.3. Color Change and Ultraviolet Barrier Properties

The color (L*, a* and b*) of the films was measured using a colorimeter (TA-XT plus 40762, Konica Minolta Sensing, Inc., Tokyo, Japan), and the UV-blocking properties of the films were evaluated using a UV spectrophotometer (UV-5500PC, Shanghai Yuan Analytical Instrument Co., Shanghai, China) in the wavelength range of 200–800 nm. The opacity of the samples was slightly modified following the method described by Zhang et al. [9] using the following Equation (1):

(1) $$p=\frac{-{log}_{10}\left(T\right)}{m}$$

Herein, p represents the opacity of the PE film sample (%), T denotes the visible light transmittance of the sample at 600 nm (%) and m is the thickness of the PE film sample (mm).

### 2.4. ATR-FTIR and XRD

The ATR-FTIR spectra of the PE film samples were obtained using a Fourier infrared spectrometer (Thermo Scientific Nicolet iS5, Grafo Industrial Equipment (Suzhou) Co., Waltham, MA, USA) for scanning. The scanning wavelength range was set from 400 cm^−1^ to 4000 cm^−1^, with 32 scans and a resolution of 4 cm^−1^. Meanwhile, the crystallinity of the samples was characterized using an X-ray diffractometer (Rigaku SmartLab, Nihon Rikaku Co., Chiba, Japan). The scanning range was set from 2θ 10° to 80°, and the scanning rate was 2°/min.

### 2.5. Microscopic Morphology

The microscopic morphology of PE composite films was observed using a scanning electron microscope (ZEISS GeminiSEM 300, Carl Zeiss Meditec, Inc., Berlin, Germany). The film samples were freeze-fractured in liquid nitrogen during the testing process. Subsequently, the samples were sprayed with gold and placed on a film disk with a diameter of 6 mm. The voltage was set to 3.0 KV. The microstructures of the samples were scanned and observed on both the surface and cross-section of the samples. Energy spectra were also scanned on the cross-section of the samples.

### 2.6. Moisture Content (MC), Water Vapor Permeability (WVP) and Water Contact Angle (WCA)

The MC of the samples was determined using a blast-drying oven. Three PE composite film samples of appropriate size were selected, and their initial weights were recorded. The samples were then dried in a blast drying oven at 105 °C for 12 h and weighed again. The MC of the film samples was calculated by dividing the difference between the initial and final weights by the initial weight.

The WVP of PE film samples was determined following a method described in previous studies with slight modifications [4]. Conical flasks containing 5 g of anhydrous calcium chloride were covered with sample films of 2.4 cm diameter and 50 mL volume. The flasks were securely sealed with waterproof tape and fixing ties. These conical flasks were placed in an environment with a temperature of 25 °C and a humidity of 70%. The flasks were weighed every 2 h for 36 h. The WVP of the film samples was calculated using the following Equation (2):

(2) $$WVP=\frac{\Delta m\times l}{\Delta p\times t\times e}$$

Herein, Δm is the weight change (g) of the conical flask during the interval time t 0(s); l, Δp and e denote the thickness of the films (m), the pressure difference between the inside and outside of the conical flask (Pa), and the penetration area of the film sample (m^2^), respectively.

In addition, the WCA of the PE film samples was determined using an interfacial tension apparatus (OSA100C, Ningbo New Boundary Scientific Instrument Co., Ningbo, China).

### 2.7. Mechanical Properties

The mechanical properties (tensile strength and elongation at break) of the PE film samples were measured using a texturometer (TA-XT plus 40762, Stable Micro Systems, Inc., Godalming, UK). The samples were first cut into strips with dimensions of 50 mm × 10 mm. The texturometer was then set with an effective length of 30 mm and a tensile rate of 1 mm/s. The samples were subjected to the tensile test to determine their tensile strength and elongation at break.

### 2.8. Antioxidant Properties and Biodegradability

The antioxidant properties of the PE film samples were determined according to the method of Zhang et al. [16] with a slight modification: First, 100 mg of film samples were dissolved in 10 mL of distilled water and then centrifuged for 10 min. Next, 1 mL of the supernatant was mixed with 3 mL of 1 mM DPPH (2,2-diphenyl-1-trinitrophenylhydrazine), and the absorbance value was measured at a wavelength of 517 nm after the mixture was incubated in water for 30 min at room temperature.

Similarly, the ABTS radical scavenging ability of pectin composite films was determined as follows [17]: Firstly, 7 mM ABTS and 2.45 mM potassium persulfate were prepared and mixed in equal proportions and then stored away from light for 16 h. Subsequently, the ABTS stock solution was diluted using ethanol until the absorbance value of the solution at 734 nm was about 0.87, to obtain the desired ABTS experimental reagent. Then, 0.1 mL of pectin composite film extract and 0.8 mL of ABTS reagent were taken and mixed and incubated under light-avoidance conditions for 10 min, and then their absorbance values were determined at 734 nm.

### 2.9. Application of PE/TA/Fe^3+^ Coating in Passion Fruit

The passion fruits were first cleaned and dried. Then, 9 passion fruits were individually coated with PE coating, PE/TA coating and PE/TA/Fe^3+^ coating. Each passion fruit was soaked in the respective coating solution for 30 s and then left to dry at room temperature. The different treatment groups were classified as control (CK), pectin coating (PE), pectin/tannic acid coating (PE/TA) and pectin/tannic acid/Fe^3+^ coating (PE/TA/Fe^3+^) based on the different treatments. The passion fruits from each treatment group were placed on the inner lid of a perforated plastic box and kept at room temperature (26 °C, 50% RH) for 10 days. The weight loss rate and degree of decay of the passion fruits were recorded daily to assess the effectiveness of the different coatings in preserving the fruit’s freshness and quality.

### 2.10. Statistical Analysis

The variables were divided into 3 groups according to the added substances, and the samples were independent of each other, with a sample size of 9 films per group, and the experiment was repeated 3 times for each group. All the experimental data obtained were analyzed using SPSS 26.0 to compare the physicochemical properties of the three groups by one-way ANOVA and Duncan’s multiple ANOVA significant difference analysis tests and are expressed as mean ± standard deviation at a 5% confidence interval. The results were close to a normal distribution, while all the experimental data images were plotted using Origin 2021. The same analytical method was used for the passion fruit coating experiments, with a sample size of 9 passion fruits per group, which were independent of each other and randomly sampled during the experiments for measurement.

## 3. Results and Discussion

### 3.1. Thickness, Color and Optical Properties of PE Composite Films

**Figure 1:**
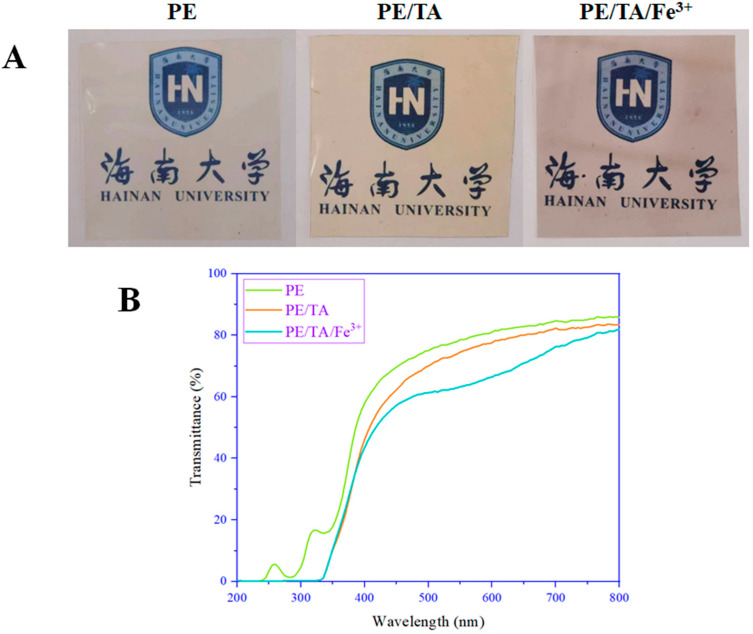
Appearance images (**A**) and UV–visible transmission spectra (**B**) of PE, PE/TA and PE/TA/Fe^3+^ films. PE: pectin, PE/TA: pectin/tannic acid, PE/TA/Fe^3+^: pectin/tannic acid/Fe^3+^.

The thickness of the PE film is the main factor influencing the barrier and mechanical properties [9,18]. Generally, the addition of TA and Fe^3+^ leads to an increase in the thickness of the PE film. As shown in Table 1, the thickness of the pure PE film was 86 μm, while with the addition of TA and Fe^3+^, the thickness of the PE film gradually increased, and the maximum thickness (97 μm) was observed in the PE/TA/Fe^3+^ film. This increase is mainly attributed to the fact that the introduction of TA and Fe^3+^ increased the solid content in the film and disrupted the original structure of the PE film. Additionally, TA is a brownish-yellow solid powder, while the Fe^3+^ solution also has a darker yellowish color, which may affect the color of the PE film when incorporated. As shown in Figure 1A, PE films doped with TA and Fe^3+^ exhibited a lighter purplish-red color with the highest a* values compared to the CK, while PE films crosslinked with TA showed a pronounced yellowness with the highest b* value. The change in the color of PE was mainly attributed to the addition of TA and Fe^3+^, indicating that TA and Fe^3+^ were uniformly dispersed in the PE film, creating a bonding interaction with the PE and resulting in a significant difference in the color of the PE film. However, the addition of TA and Fe^3+^ also resulted in an increase in the transparency of the PE film. The PE composite film still exhibited a colorless and transparent appearance with a visible light transmittance of more than 60%. This demonstrates that the addition of TA and Fe^3+^ does not affect consumers’ visual judgment of the appearance of food products and could be beneficial for the commercial sale of food products. As shown in Figure 1B, the pure PE film has a transmittance of 1–18% in the range of 280–350 nm, while the PE film containing TA and Fe^3+^ shows a transmittance near 0%. This is mainly due to the strong UV absorption of TA and the excellent UV-blocking properties of Fe^3+^. It has a high potential for the preservation and protection of some UV-sensitive and photo-oxidative rancidity-susceptible food products. Some studies have reported similar phenomena, such as incorporating TA into sodium alginate films, resulting in sodium alginate films showing 0% transmittance in the UV region of 200–320 nm [19]. Another study reported that 1.6% Fe^3+^-crosslinked sodium alginate films showed 100% UV shielding at 200–280 nm [20]. Importantly, the light absorption of Fe^3+^ is located in the near-ultraviolet part of the electromagnetic spectrum, and the UV absorption function of Fe^3+^ determines the strength of its UV-blocking properties [21].

### 3.2. FTIR and XRD Analysis

**Figure 2:**
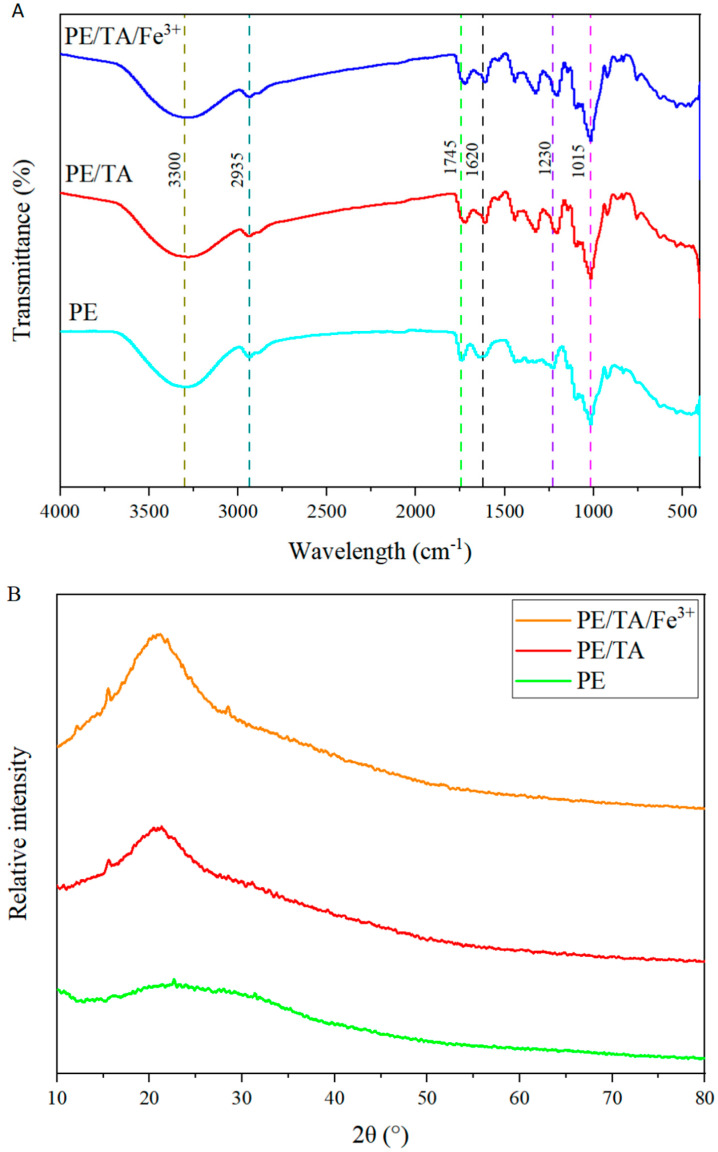
FTIR (**A**) and X_crystal diffraction structure (**B**) analysis of PE, PE/TA and PE/TA/Fe^3+^ films. PE: pectin, PE/TA: pectin/tannic acid, PE/TA/Fe^3+^: pectin/tannic acid/Fe^3+^.

The FTIR spectra of PE composite films are shown in Figure 2A. The pure PE films show broader bands at 3300 cm^−1^, which can be attributed to the hydroxyl groups present in the PE molecules, glycerol and water molecules, triggering O-H stretching vibrations [22]. With the addition of TA and Fe^3+^, the intensity of the broad peaks of the PE films decreases slightly, and the frequency band broadens. This is mainly due to the crosslinking of PE with TA and Fe^3+^, resulting in the rearrangement of PE molecules and a significant decrease in the hydroxyl interactions between PE and TA [9]. Similarly, a study reported that the hydroxyl group of lemon peel PE can interact with purple sugarcane peel anthocyanin extract to form a hydrogen bond, which, in turn, reduces the intensity of the corresponding O-H peak [16]. All PE films show a peak signal at 2935 cm^−1^, which may be related to the vibration of the CH_2_ group in the PE molecule [23]. It is noteworthy that the characteristic spectral peaks at 1735 cm^−1^ and 1620 cm^−1^ of the PE films are derived from the methylated carboxylate and unmethylated carboxylate groups of the PE. It can be observed that the peaks of the methylated carboxylate groups are significantly higher than those of the unmethylated carboxylate groups, indicating a higher esterification degree of the PE (>50%), which is classified as highly methoxylated PE [24]. Meanwhile, the PE films show stretching vibrations of C-O and C-O-C bonds at 1230 cm^−1^ and 1015 cm^−1^, respectively. Thus, the study in this work found no significant changes in the FTIR of PE composite films doped with TA and Fe^3+^ compared to the pure PE films and no new bands of spectral features, indicating the ability of TA and Fe^3+^ to be homogeneously dispersed in the PE films and not create new chemical interactions. This result is in agreement with the study of Zhao et al. [25]. Furthermore, Figure 2B shows the X_crystal diffraction structure of PE films. The pure PE film exhibits a broader diffraction peak at 2θ = 20.88°, indicating the presence of an amorphous structure in the PE film. While the position of the broad peak at 2θ = 20.88° of the PE film does not change with the addition of TA and Fe^3+^, the peak width narrows. This indicates the possible interaction of PE with TA and Fe^3+^, which improves the crystallinity of PE films without changing the amorphous structure of PE. This phenomenon also illustrates the ability of tannins and Fe^3+^ to be uniformly dispersed in PE films with excellent biocompatibility among the three [8]. Muhoza et al. [26] reported that crosslinking PE and gelatin films using TA increased the crystalline strength of the films by increasing the helices of gelatin but did not change the position of the broad peaks, illustrating the ability of TA to homogeneously dope gelatin and PE composite films. This is similar to the results of this work.

### 3.3. Micromorphology of PE Composite Films

**Figure 3:**
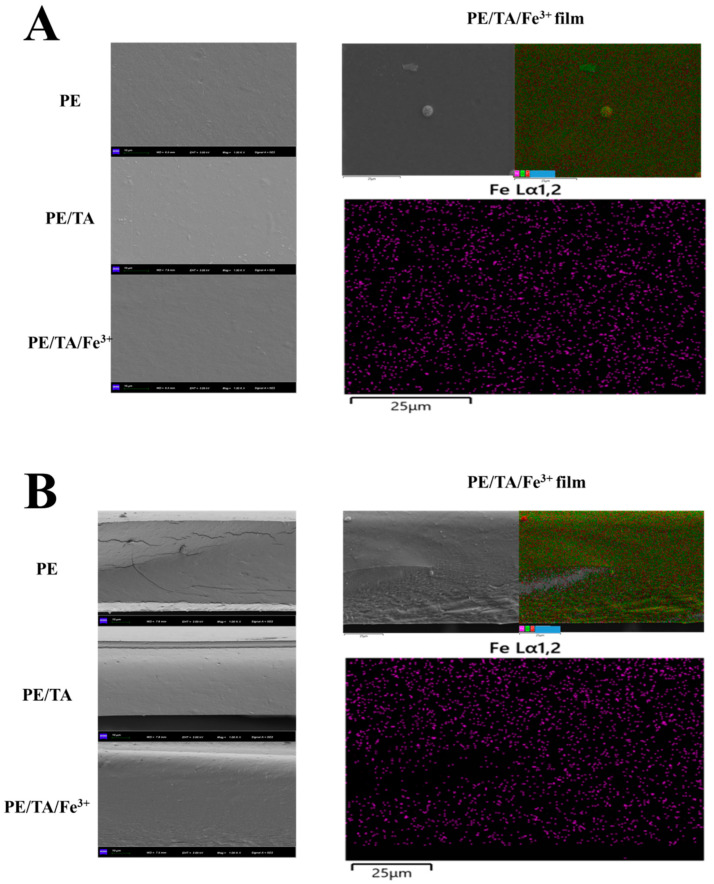
Surface microstructure (**A**) and cross-section microstructure (**B**) of PE, PE/TA and PE/TA/Fe^3+^ films. The magnification of the surface and cross-section microstructures of pectin composite films was ×1500 and ×1000, respectively. PE: pectin, PE/TA: pectin/tannic acid, PE/TA/Fe^3+^: pectin/tannic acid/Fe^3+^.

**Figure 4:**
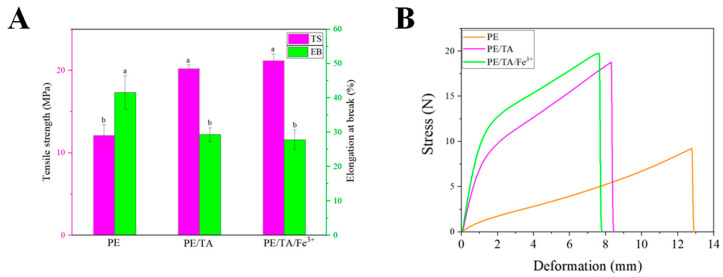
Tensile strength, elongation at break (**A**) and stress–strain curve (**B**) of PE, PE/TA and PE/TA/Fe^3+^ films. PE: pectin, PE/TA: pectin/tannic acid, PE/TA/Fe^3+^: pectin/tannic acid/Fe^3+^. Superscript letters with the same column represent the test of significance difference between the data of each treatment group, letters with the same letter indicate that there is no significant difference in Duncan’s Multiple Ranges (*p* > 0.05).

The microstructure of the PE film was mainly analyzed and observed by scanning electron microscopy (SEM), as shown in Figure 3A, which presents the surface microstructure of the PE composite film at 1500× magnification. All PE films exhibit a relatively flat and uniform surface microstructure due to the excellent film-forming properties of PE. Figure 3B shows the cross-sectional microstructure of the PE films. At 1000× magnification, a large number of cracks and voids were observed in the cross-sectional microstructure of the pure PE films. However, with the addition of TA and Fe^3+^, the voids and cracks in the PE films were significantly reduced, and a continuous, dense and flat cross-sectional structure was observed in the PE/TA/Fe^3+^ films. This indicates that TA and Fe^3+^ are capable of crosslinking with each other and improving the loose structure between PE molecules. Specifically, TA not only crosslinks with PE molecules but also further reduces the discontinuous molecular arrangement of PE through the metal–phenol supramolecular network structure with Fe^3+^, which significantly improves the degree of crosslinking between PE molecules, resulting in the formation of a dense and compact network structure. Similarly, another study observed that the loose and porous cross-sectional structure of PE films became dense and homogeneous when PE films were crosslinked with Fe^3+^ [9]. Additionally, the surface and cross-sectional microstructures of PE in Figure 4 show that Fe^3+^ is uniformly distributed in the PE film, indicating that Fe^3+^ and PE molecules are uniformly dispersed inside the PE film through crosslinking with each other. This phenomenon is consistent with the study of Bai et al. [19].

### 3.4. MC, WVP and WCA of Pectin Composite Films

The moisture content (MC), water vapor permeability (WVP) and water contact angle (WCA) of the PE films are presented in Table 2. The MC of the pure PE film was 18.57 ± 0.26%, while the incorporation of TA resulted in a 25.8% decrease in the MC of the PE film. This decrease can be attributed to the higher hydrophobicity of tannins compared to PE polymers. Similarly, a study found that the doping of hydrophobic nanosilver ions into PE films also led to a reduction in their MC from 9.4% to 8.6% [27]. However, the MC of the PE films slightly increased with the incorporation of Fe^3+^, mainly due to the formation of a metal–phenol supramolecular network structure between Fe^3+^ and TA. This network structure reduces the interaction between TA and PE, resulting in an increase in the MC of the PE films. Therefore, the hydrophobicity and interaction of the added components significantly affect the water content. On the other hand, WVP is an important parameter for evaluating the barrier properties of water molecules. As shown in Table 2, the pure PE film exhibited the highest WVP of 1.61 ± 0.06 × 10^−10^ gm^−1^s^−1^Pa^−1^. Previous studies have shown that PE films with higher WVP have reduced water molecule barrier properties, leading to accelerated water loss from preserved foods. The WVP of PE/TA and PE/TA/Fe^3+^ films decreased by 15.5% and 23.6%, respectively, compared to the pure PE films. However, there was no significant difference between the WVP of PE/TA and PE/TA/Fe^3+^ films, indicating that the addition of TA and Fe^3+^ could reduce the WVP of the PE films. This reduction in WVP can be attributed to the crosslinking between TA and PE, which makes the network structure within the PE films denser, resulting in a decrease in the WVP of PE/TA films. Additionally, Fe^3+^ not only crosslinks with PE but also reacts with TA, further improving the density and crosslinking degree of the PE films, leading to the lowest WVP [28,29]. Similarly, Zhang et al. [9] also found that the addition of Fe^3+^ significantly reduced the WVP of PE films when different metal cations were doped into the PE films. The authors explained that the metal cations can crosslink with PE, and the higher the crosslinking, the more tortuous the path for water molecules to pass through the films, resulting in a decrease in the WVP of the PE films. In addition, the WCA is an important indicator for assessing the hydrophilicity of food films. Generally, a WCA > 65° is considered highly hydrophilic [30]. However, the WCAs of the prepared PE films were below 60°. This is mainly due to the enhanced adhesion and wettability of the network structure formed by TA and Fe^3+^.

### 3.5. Mechanical Properties

Tensile strength and elongation at break are well-known indicators for evaluating the mechanical properties of food packaging films and are highly correlated with film structure. Figure 4A presents the tensile strength and elongation at break of pure PE films and PE composite films, while the corresponding stress–strain curves are shown in Figure 4B. It can be observed that among the pure PE films, the tensile strength is the lowest, measuring 12.103 MPa, while the elongation at break is the highest, at 41.59%. With the addition of TA and Fe^3+^, the mechanical properties of the PE films were significantly improved. The tensile strength of the PE/TA film and PE/TA/Fe^3+^ film improved by 40.14% and 42.85%, respectively, compared to the pure PE film. This improvement can be mainly attributed to the ability of tannins to crosslink with PE molecules, resulting in a denser structure of the PE films. Additionally, Fe^3+^ can form a tight metal–phenol network structure with TA and crosslink with PE molecules, forming an “egg carton” model that significantly enhances the mechanical properties of the PE films [9]. The microstructure of the PE films also confirms that the addition of TA and Fe^3+^ leads to a more compact and dense structure of PE. Similarly, Zhao et al. [4] reported that the tensile strength of films could be significantly improved by incorporating TA into chitosan–corn starch films. The authors explained that TA can react with chitosan molecules in a Schiff base reaction, forming covalent bonds that enhance the rigid structure of the film. Additionally, intermolecular forces such as hydrogen bonding interactions and electrostatic interactions that may exist between PE molecules and TA could also be important in improving the mechanical properties of the PE films.

### 3.6. Antioxidant Properties

**Figure 5:**
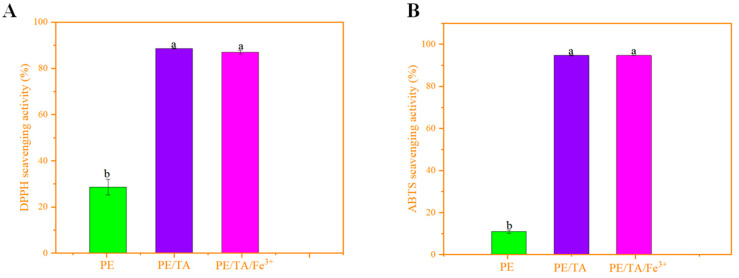
DPPH scavenging capacity (**A**) and ABTS scavenging capacity (**B**) of PE, PE/TA and PE/TA/Fe^3+^ films. PE: pectin, PE/TA: pectin/tannic acid, PE/TA/Fe^3+^: pectin/tannic acid/Fe^3+^. Superscript letters with the same column represent the test of significance difference between the data of each treatment group, letters with the same letter indicate that there is no significant difference in Duncan’s Multiple Ranges (*p* > 0.05).

As depicted in Figure 5A, pure PE films exhibited the lowest antioxidant activity at 28.59%. However, the addition of TA and Fe^3+^ significantly improved the antioxidant activity of the PE films. Specifically, the DPPH radical scavenging activity of the PE/TA composite film increased to 88.6%, while the combined addition of Fe^3+^ and TA slightly reduced the antioxidant activity to 86.97%. However, despite the advantages of rapidity, efficiency and robustness of DPPH radical scavenging rate determination, according to Christodoulou et al. [31], the determination for hydrophilic substances may be affected by a number of interferences and artifacts resulting in absorption of the tested substrate and antioxidant substances by the same light region, which ultimately influences the results of the experiments. Therefore, the pectin composite films were also studied using the ABTS reagent assay as ABTS detects hydrophilic and lipophilic antioxidant substances, further confirming the antioxidant capacity of the pectin composite films.

As shown in Figure 5B, the ABTS radical scavenging rate of pure pectin films was 10.93%, whereas the antioxidant activity of pectin films increased by about 88.47% with the addition of TA. This confirms that the introduction of TA significantly enhanced the antioxidant capacity of pectin films. However, the addition of Fe^3+^ slightly reduced the antioxidant activity of tannins in pectin films. This observation can be explained by the properties of TA. TA contains hydroxyl groups and is known for its potent antioxidant activity [32]. The hydroxyl groups in TA contribute to its antioxidant properties. However, Fe^3+^ reacts with the phenolic hydroxyl groups in TA, forming a metal–phenolic network, which reduces the number of available hydroxyl groups in TA. As a result, the DPPH scavenging activity of the PE composite film decreases. Another study found that encapsulating 2% TA in an ultrafine cellulose acetate fiber mat using the electrostatic spinning technique increased the antioxidant activity by 41% [33]. Additionally, the co-doping of Fe^3+^ with TA decreased the antioxidant activity, but there was no significant difference compared to the antioxidant activity of 2% TA alone. This can be attributed to the lower content of Fe^3+^, which retains the strong antioxidant capacity of TA despite the binding affinity between TA and Fe^3+^ and the reduction in its hydroxyl content [34]. It is noteworthy that both assays for antioxidant activity were performed using a spectrophotometer. Although a spectrophotometer has the advantages of being easy to use and having a low cost and high efficiency, the pH of the solution, the reaction temperature, the reaction products and the color of the substrate all affect the ultimate results.

### 3.7. Application of PE/TA/Fe^3+^ Coating in Passion Fruit

**Figure 6:**
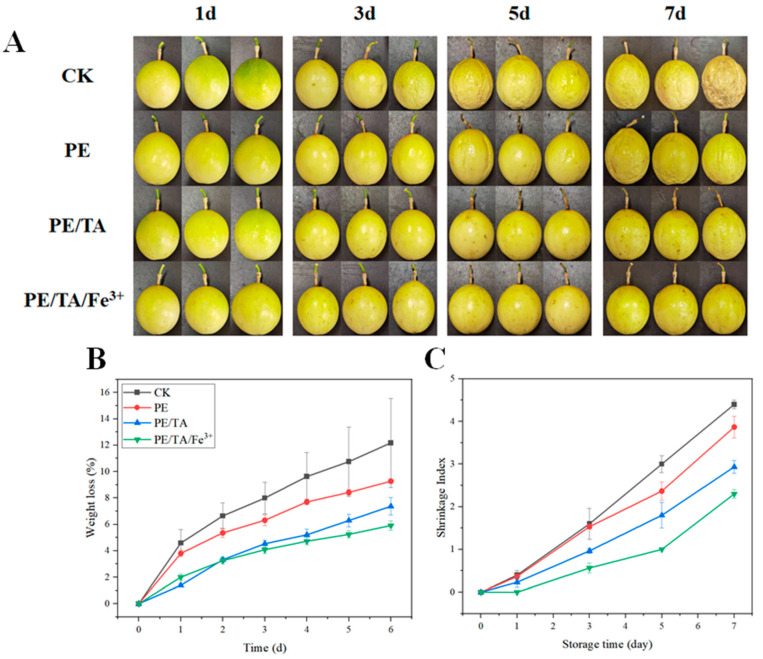
Photographs of the appearance (**A**), weight loss (**B**) and shrinkage index (**C**) of untreated and pectin composite coating-treated passion fruits over 7 days. PE: pectin, PE/TA: pectin/tannic acid, PE/TA/Fe^3+^: pectin/tannic acid/Fe^3+^.

It is well known that passion fruit is a typical climacteric fruit [35]. After harvesting, passion fruit experiences accelerated water loss and rapid skin wrinkling. The change in the appearance of passion fruit over a period of 7 days is shown in Figure 6A. In the control group (CK), obvious wrinkling appeared on the 3rd day, and with time, the wrinkling of the passion fruit worsened until the 7th day when the fruit in the CK group became rotten, deteriorated and unfit for consumption. In contrast, the appearance of passion fruit treated with the PE composite coating was significantly improved. Passion fruit treated with pure PE coating showed wrinkles on the 5th day, the PE/TA composite coating showed wrinkles on the 7th day, and the PE/TA/Fe^3+^ composite coating showed the best effect, with minimal or no wrinkles observed during the 7-day period. Additionally, the weight loss of passion fruit increased linearly during the 7-day storage period. As shown in Figure 6B, the untreated passion fruit had the highest weight loss of about 12.17%, whereas the passion fruit treated with the PE composite coating exhibited significantly lower weight loss. The passion fruit treated with the PE/TA/Fe^3+^ composite coating showed the lowest weight loss at approximately 5.98%. This observation is predictable since the PE/TA/Fe^3+^ composite coating has the lowest water vapor permeability (WVP), providing excellent barrier properties and minimizing water loss, thus maintaining the quality of the passion fruit [36]. Similarly, Zhou et al. [15] treated purple-skinned passion fruit with a chitosan/TA composite coating, and the coated fruit showed an 8.03% reduction in weight loss after 21 days of storage compared to the control group. The shrinkage index of passion fruit is also an important indicator for evaluating its quality. As shown in Figure 6C, the untreated passion fruit exhibited a sharp increase in shrinkage index during the 7-day period. Passion fruit treated with the PE composite coating showed a significantly lower shrinkage index compared to the control group. There was no significant difference in the wrinkling index between passion fruit treated with the pure PE coating and passion fruit treated with the PE/TA composite coating in the first 3 days. However, the pure-PE-coated passion fruit gradually developed wrinkles over time and showed wrinkling and black spots after 7 days of storage. Notably, passion fruits treated with the PE/TA/Fe^3+^ composite coating retained good fruit quality after 7 days of storage and had the lowest wrinkling index, indicating they were still of some food value. This result aligns with the study conducted by Zhou et al. [15]. The PE/TA/Fe^3+^ coating could influence the anaerobic respiration and fruit metabolism inside the passion fruit. Therefore, the PE composite coating treatment significantly delays the senescence and spoilage of passion fruit, improving its appearance quality and commercial value.

## 4. Conclusions

In this study, the effects of TA and Fe^3+^ multi-crosslinking systems on the properties of PE films were investigated. TA and Fe^3+^ could be uniformly doped into the PE films, which significantly improved the UV barrier properties of the PE films through crosslinking and resulted in a reduction in the WVP of the PE films of about 23.6%. This could be confirmed by FTIR, and the microstructure of the films became more compact and continuous. Meanwhile, TA and Fe^3+^ also led to an increase in the TS of PE films by 40.14% and 42.85%, respectively. The ability of tannins and Fe^3+^ to produce multiple crosslinks with PE, as analyzed by X_crystal diffraction and FTIR, indicated a favorable biocompatibility between the three. Furthermore, this work showed that the addition of TA significantly enhanced the antioxidant properties of the PE composite film, resulting in an increase in its free radical scavenging capacity of about 63.01%. It is important to note that passion fruit treated with PE composite coating had good appearance quality. Moreover, passion fruit treated with PE/TA/Fe^3+^ composite coating showed the lowest weight loss and crumpling index, indicating that the addition of TA and Fe^3+^ significantly improved the freshness preservation effect of PE coating on passion fruit. All in all, this work demonstrates that the physicochemical properties of PE films can be significantly improved through the formation of multiple crosslinks between TA and Fe^3+^. The study also highlights that PE/TA/Fe^3+^ coatings offer a promising strategy for maintaining postharvest fruit quality.

## Figures and Tables

**Table 1:** Thickness, color and opacity of PE, PE/TA and PE/TA/Fe^3+^ films. PE: pectin, PE/TA: pectin/tannic acid, PE/TA/Fe^3+^: pectin/tannic acid/Fe^3+^.

Film	Thickness (μm)	L*	a*	b*	Opacity
PE	86 ± 0.37 ^c^	86.99 ± 3.91 ^a^	0.32 ± 0.17 ^b^	0.63 ± 1.52 ^b^	1.049 ± 0.027 ^b^
PE/TA	92 ± 0.17 ^b^	74.33 ± 5.67 ^b^	0.15 ± 0.28 ^b^	6.45 ± 1.89 ^a^	1.129 ± 0.066 ^b^
PE/TA/Fe^3+^	97 ± 0.78 ^a^	68.80 ± 1.97 ^c^	3.63 ± 0.74 ^a^	1.02 ± 1.07 ^b^	1.749 ± 0.077 ^a^

Values with the same letter in the same column are shown as not significantly different according to Duncan’s multiple range test (*p* < 0.05). Data are shown as mean ± standard error (*n* ≥ 9).

**Table 2:** Moisture content, water vapor permeability and water contact angle of PE, PE/TA and PE/TA/Fe^3+^ films. PE: pectin, PE/TA: pectin/tannic acid, PE/TA/Fe^3+^: pectin/tannic acid/Fe^3+^.

Film	WC (%)	WVP (×10^−10^ gm^−1^s^−1^Pa^−1^)	WCA (°)
PE	18.57 ± 0.26 ^a^	1.61 ± 0.06 ^a^	52.73 ± 3.23 ^a^
PE/TA	13.78 ± 0.32 ^b^	1.36 ± 0.08 ^b^	30.36 ± 2.48 ^b^
PE/TA/Fe^3+^	14.42 ± 0.44 ^b^	1.23 ± 0.14 ^b^	17.92 ± 1.66 ^c^

Values with the same letter in the same column are shown as not significantly different according to Duncan’s multiple range test (*p* < 0.05). Data are shown as mean ± standard error (*n* ≥ 3).

## Data Availability

Data is contained within the article.
